# Frequency Optimization for Enhancement of Surface Defect Classification Using the Eddy Current Technique

**DOI:** 10.3390/s16050649

**Published:** 2016-05-07

**Authors:** Mengbao Fan, Qi Wang, Binghua Cao, Bo Ye, Ali Imam Sunny, Guiyun Tian

**Affiliations:** 1 School of Mechatronic Engineering, China University of Mining and Technology, Xuzhou 221116, China; wuzhi3495@cumt.edu.cn; 2 Jiangsu Key Laboratory of Mine Mechanical and Electrical Equipment, China University of Mining and Technology, Xuzhou 221116, China; 3 School of Information and Electrical Engineering, China University of Mining and Technology, Xuzhou 221116, China; caobinghua@cumt.edu.cn; 4 Faculty of Electric Power Engineering, Kunming University of Science and Technology, Kunming 650500, China; yeripple@hotmail.com; 5 School of Electrical and Electronic Engineering, Newcastle University, Newcastle Upon Tyne NE1 7RU, UK; a.sunny@newcastle.ac.uk (A.I.S.); g.y.tian@newcastle.ac.uk (G.T.)

**Keywords:** nondestructive testing, eddy current sensor, frequency optimization, probe response, feature extraction, defect classification

## Abstract

Eddy current testing is quite a popular non-contact and cost-effective method for nondestructive evaluation of product quality and structural integrity. Excitation frequency is one of the key performance factors for defect characterization. In the literature, there are many interesting papers dealing with wide spectral content and optimal frequency in terms of detection sensitivity. However, research activity on frequency optimization with respect to characterization performances is lacking. In this paper, an investigation into optimum excitation frequency has been conducted to enhance surface defect classification performance. The influences of excitation frequency for a group of defects were revealed in terms of detection sensitivity, contrast between defect features, and classification accuracy using kernel principal component analysis (KPCA) and a support vector machine (SVM). It is observed that probe signals are the most sensitive on the whole for a group of defects when excitation frequency is set near the frequency at which maximum probe signals are retrieved for the largest defect. After the use of KPCA, the margins between the defect features are optimum from the perspective of the SVM, which adopts optimal hyperplanes for structure risk minimization. As a result, the best classification accuracy is obtained. The main contribution is that the influences of excitation frequency on defect characterization are interpreted, and experiment-based procedures are proposed to determine the optimal excitation frequency for a group of defects rather than a single defect with respect to optimal characterization performances.

## 1. Introduction

Defects may occur in the stress concentration areas of in-service components or systems. Generated defects gradually grow due to stress and pose a severe threat to the structural integrity of in-service parts or systems [1,2,3]. Therefore, it is essential that periodic inspection be implemented nondestructively for safe and efficient operation. Non-destructive testing (NDT) is an invaluable tool to detect, identify and characterize defects in a component or system without hindering its future usability. Compared to common ultrasonic [4,5] and X-ray [6,7] techniques, eddy current testing (ECT), which operates on the principles of electromagnetic induction, has many advantages such as its efficiency, low cost, noncontact nature, implementation and so on. Currently, ECT has been widely accepted as a desirable technique for characterization of defects in metallic parts [8,9,10].

Characterization of a defect is to find a solution to an inverse problem, and has to be completed before evaluation and decision-making concerning maintenance and replacement [11]. In many cases, the relationship between defects and probe signals is very complex and nonlinear. Although considerable effort has been devoted to characterization of defects for decades, this subject still remains a hot topic owing to ill-posedness and a variety of practical cases [11,12]. Most inversion methods for nondestructive evaluation (NDE) could basically be classified either as model-based or model-free [13]. The model-based method takes characterization of defects as an optimization problem. It requires an accurate and efficient forward model, and could deal with complex defects [13]. However, a model-based method suffers from a heavy computational burden because of the iterative operation of a forward model [14]. Conversely, a model-free method normally adopts signal processing and machine learning algorithms to solve NDE inverse problems. The key to a model-free method is to mathematically relate extracted features from probe responses to defect parameters using a set of training data from defect samples. Compared to model-based methods, model-free methods are more efficient and popular [11].

There are many factors that affect the performance of model-free based characterization of defects. Extensive investigations have been conducted to improve defect characterization with regard to reduction of liftoff effect, new design and optimization of probes, and feature extraction [11,15]. The liftoff effect, which is the variation of the distance between the probe and the specimen, produces unwanted noise and degrades probe signals significantly. It is one of the main obstacles for effective ECT [16]. Tian *et al.* [17] normalized probe signals to suppress the liftoff effect for pulsed eddy current (PEC) evaluation. Yu *et al.* [18] theoretically and experimentally investigated the relationship between peak value of PEC response and liftoff distance for reduction of liftoff noise. Novel design and optimization of probe in the configuration and parameters dedicated to a specific case is another way to enhance defect evaluation [19,20,21]. Joubert *et al.* [22] fabricated an array probe to acquire high spatial resolution images of sub-millimetric surface defects. Rosado *et al.* [23] evaluated the influences of geometrical parameters of a planar probe towards signal amplitude and spatial discrimination in a bid to improve defect characterization. Recently, advanced signal processing methods were employed to increase the contrast between features of different defects [24,25]. Principal component analysis (PCA) is the most popular feature extraction method. Nevertheless, PCA cannot guarantee the statistical independence of derived principal components [26]. To overcome the drawbacks of PCA, independent component analysis (ICA) is proposed to extract defect features. It is noted that both PCA and ICA are linear methods in nature, thus they are incapable of handling nonlinear problems [27]. Kernel PCA (KPCA), which is regarded as an extended PCA, is developed by constructing a nonlinear mapping from input space to high-dimensional space with a kernel function. Extensive reports confirm that KPCA is superior to PCA [27].

In addition to the aforementioned factors, excitation strategy plays an important role in defect characterization as well. At present, single frequency, multiple frequency and pulsed excitation are presented to acquire more information on defects [24,28,29]. However, the influences of excitation frequency on defect characterization have not been paid much attention yet. A few reports show that maximum probe signals were observed by tuning frequency for a single specific defect, which is recommended to enhance the NDE of structural integrity [30,31,32,33,34]. Unfortunately, the findings in [30,31,32,33] were only restricted to a single defect, and not suitable for a set of defects. In different industries, the defects to be identified are generally different in size. Therefore, there is a demand to find an optimal frequency for a group of defects. Moreover, the influences of excitation frequency should be fully investigated in terms of detection sensitivity, contrast between defect features and classification accuracy, which are to be exploited.

The purpose of this paper is to enhance surface defect classification by seeking the optimal excitation frequency. In order to determine the optimal frequency, the influences of excitation frequency on characterization performances for a set of defects were disclosed and interpreted in terms of detection sensitivity, contrast between extracted features and accuracy of classification. The main contribution of this work lies in the experiment-based procedures for determining the optimal frequency applied to a group of defects in terms of characterization performances. The remainder of this work is arranged as follows: KPCA for feature extraction and support vector machine (SVM) for classification of defects are briefly introduced in Section 2. Then, the experimental setup and fabricated specimen are described in Section 3. In Section 4, the influences on probe sensitivity, contrast between extracted features and misclassification are analyzed to derive the optimal excitation frequency so as to enhance characterization of surface defects. Finally, conclusions and further work are outlined in Section 5.

## 2. Methodology

### 2.1. Kernel PCA

PCA is a statistical multivariate analysis technique. It transforms original observed data into a new set of uncorrelated variables. Each derived variable termed as a principal component is a linear transformation of original observed data. In theory, KPCA is a nonlinear extension of standard PCA using kernel methods [35]. As a result, KPCA is well suited to extract interesting nonlinear structures [36].

Assuming there is a set of data sampled from probe signals corresponding to a given scanning route $x_{i},\;i=1,...,N,\;x_{i}\in R^{M}$. Suppose we map the data ***x****_i_* into a feature space ***F*** using a nonlinear transformation ϕ(x)

(1) $$\phi :\;R^{M}\to F,\;x\to X$$

Given that the data $\phi (x_{i})$ mapped into the feature space ***F*** are centered, *i.e.*, $\frac{1}{N}\sum_{i=1}^{N}\phi (x_{i})=0$, to perform PCA, the covariance matrix ***C*** in the space ***F*** is calculated by

(2) $$C=\frac{1}{N}\sum_{i=1}^{N}\phi (x_{i})\phi {\left(x_{i}\right)}^{\text{T}}$$

We need to find Eigenvalues and Eigenvectors satisfying

(3) $$\lambda_{k}v_{k}=Cv_{k},\;k=1,2,...,M$$

where λ*_k_* and ***v****_k_* stand for non-zero eigenvalue and eigenvector of the covariance matrix ***C***, respectively.

Substituting Equation (3) into Equation (2), we have

(4) $$\frac{1}{N}\sum_{i=1}^{N}\phi (x_{i})\{\phi {\left(x_{i}\right)}^{T}v_{k}\}=\lambda_{k}v_{k}$$

and there exists a coefficient $\alpha_{i}$ such that

(5) $$v_{k}=\sum_{i=1}^{N}\alpha_{ki}\phi (x_{i})$$

From Equations (4) and (5), we have

(6) $$\frac{1}{N}\sum_{i=1}^{N}\phi (x_{i})\phi {\left(x_{i}\right)}^{T}\sum_{j=1}^{N}\alpha_{kj}\phi (x_{j})=\lambda_{k}\sum_{i=1}^{N}\alpha_{ki}\phi (x_{i})$$

Defining the kernel function $\kappa (x_{i},x_{j})=\phi {\left(x_{i}\right)}^{T}\phi (x_{j})$ and kernel matrix $K_{i,j}=\kappa (x_{i},x_{j})$, Equation (6) can be simplified by means of matrix notation as

(7) $$K\alpha_{k}={\overset{-}{\lambda }}_{k}\alpha_{k}$$

where ${\overset{-}{\lambda }}_{k}$ and $\alpha_{k}$ are the eigenvalue and eigenvector of the kernel matrix ***K***, respectively.

Finally, the resulting kernel principal components can be calculated using

(8) $$y_{k}(x)=\phi {\left(x\right)}^{T}v_{k}=\sum_{i=1}^{N}\alpha_{ki}\kappa (x,x_{i})$$

The elegance of using the kernel matrix ***K*** is that we can cope with $\phi (x_{i})$ of arbitrary dimensionality without having to compute $\phi (x_{i})$ explicitly [36].

### 2.2. Support Vector Machine

The classification problem can be restricted to consideration of the two-class problem without loss of generality. In this application, the goal is to produce a classifier that will generalize well in unknown cases. The support vector machine (SVM), founded by V. Vapniks, is a pattern recognition technique based on structural risk instead of empirical risk minimization. Therefore, an SVM has better generalization capabilities, and suitable for small sample size.

Given a dataset $S=\{(x_{i},y_{i})\}$, i=1,2,⋯,N, where $x_{i}\in R^{d}$ and $y_{i}\in \{-1,1\}$ denote the input and output of a classifier, respectively. Theoretically, there are multiple linear classifiers that can separate the data in ***S*** correctly. However, only the optimal hyperplane in canonical form can maximize the margin between the two classes, which is formulated as

(9) $$y_{i}[w\cdot x+b]\geq 1,\;i=1,...,N$$

The margin could be derived by

(10) $$\rho (w,b)=\frac{2}{\left\|w\right\|}$$

Hence, the optimal hyperplane is determined by minimizing the following function with the constraints of Equation (9)

(11) $$\Phi (w)=\frac{1}{2}{\left\|w\right\|}^{2}$$

To solve Equation (11), we define the Lagrange function below

(12) $$L(w,b,\alpha )=\frac{1}{2}{\left\|w\right\|}^{2}-\sum_{i=1}^{N}\alpha_{i}(y_{i}[w\cdot x_{i}+b]-1)$$

where $\alpha_{i}$ is the Lagrange multiplier.

$\alpha_{i}$ is given by minimizing

(13) $$\min\frac{1}{2}\sum_{i=1}^{N}\sum_{j=1}^{N}y_{i}y_{j}\alpha_{i}\alpha_{j}\langle x_{i},x_{j}\rangle -\sum_{j=1}^{N}\alpha_{j}$$

with constraints,

(14) $$\alpha_{i}\geq 0,\;\mathrm{and}\;\sum_{i=1}^{N}\alpha_{i}y_{i}=0$$

The aforementioned formulation of the optimal hyperplane is restricted to linearly separable cases. However, this will not be the case in general. To deal with linearly non-separable cases, a kernel function and penalty factor *C* are introduced. A kernel function is adopted to map observed data into a high-dimensional feature space in order to transform a linearly non-separable case to a linearly separable case. As a result, Equations (13) and (14) become

(15) $$\min\frac{1}{2}\sum_{i=1}^{N}\sum_{j=1}^{N}y_{i}y_{j}\alpha_{i}\alpha_{j}\kappa (x_{i},x_{j})-\sum_{j=1}^{N}\alpha_{j}$$

subject to the constraints

(16) $$0\leq \alpha_{i}\leq C,\;\mathrm{and}\;\sum_{i=1}^{N}\alpha_{i}y_{i}=0$$

where *C* is a penalty factor, and $\kappa (x_{i},x_{j})$ is a kernel function.

After the variable α*_i_* is obtained, the decision function used to classify sampled dataset from sensors is

(17) $$y(x)=\mathrm{sign}(\sum_{i=1}^{n}\alpha_{i}y_{i}\kappa (x_{i}\cdot x)+b)$$

## 3. Experimental Setup and Specimens

**Figure 1:**
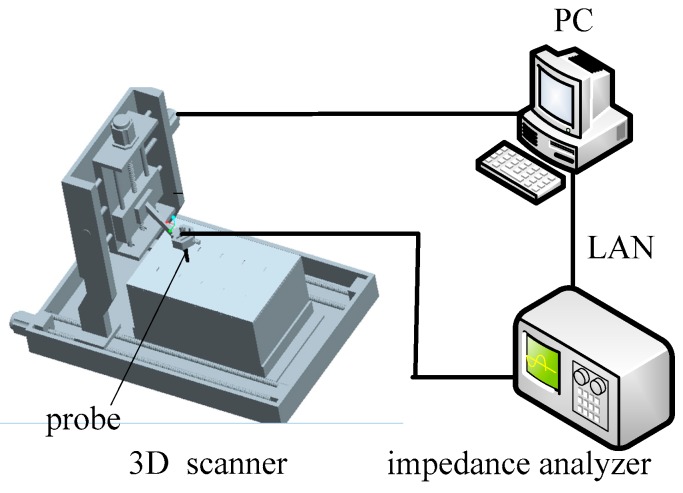
Automated experimental setup.

The automated experimental setup developed for eddy current characterization of defects is composed of an impedance analyzer, a probe, a 3D scanner and a PC, as shown in Figure 1. The probe is a packaged air-cored coil. It has an inner diameter of 4.0 mm, outer diameter of 4.8 mm, height of 5.0 mm and 300 turns, respectively. The WK65120B impedance analyzer (Wayne Kerr Electronics, West Sussex, UK) which nominally has a broad bandwidth ranging from 20 Hz to 120 MHz and 0.05% basic measurement accuracy, is used to obtain the impedance of the probe. The PC acquires probe signals from the impedance analyzer through the LAN. The 3D scanner is controlled by pulse width modulation (PWM) signals from a PCI-bus card MPC08 with an application developed in Labview (Leetro Automation Co., Ltd., Chengdu, China) and installed in the PC. A pulse from PWM signals can drive a step motor to rotate a fixed angle. Therefore, the displacement and the speed of the 3D scanner are proportional to the number and frequency of the pulses fed into the motor, respectively.

**Figure 2:**
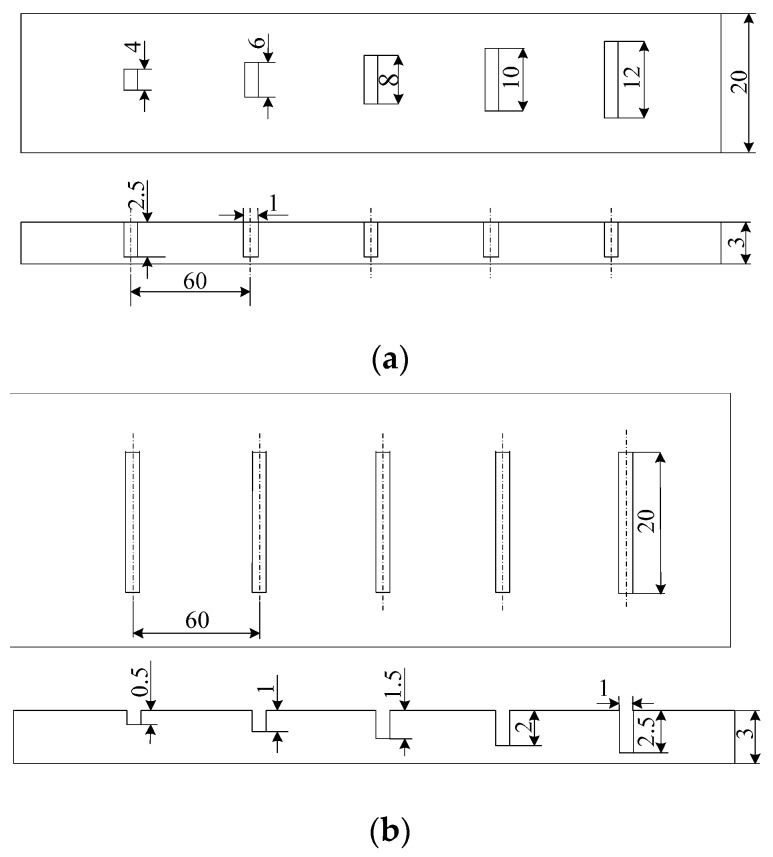
Fabricated specimens. (**a**) Sample 1: Defects with different lengths; (**b**) Sample 2: Defects with different depths.

Two Al alloy specimens were prepared, and ten artificial slots were manufactured by electrical discharge machining (EDM) for training and test datasets, as illustrated in Figure 2. Geometrical parameters of the manufactured defects are listed in Table 1. Both of the samples have a thickness of 3.0 mm. The defects in sample 1 have a constant depth of 2.5 mm and lengths with values 4.0, 6.0, 8.0, 10.0 and 12.0 mm. The defects in sample 2 have depths ranging from 0.5 mm to 2.5 mm in steps of 0.5 mm with a constant length of 20.0 mm. All the fabricated defects have a uniform width of 1.0 mm.

## 4. Results and Discussion

In this section, we will experimentally assess the influences of excitation frequency on the classification of surface defects in light of detection sensitivity, dissimilarity of defect features and classification accuracy. As the probe is fixed on the 3D scanner, the liftoff distance is kept constant during inspection. The probe moves in a direction perpendicular to the defects. After multiple trials, scanning distance is set to 10 mm with a step of 0.1 mm, and scanning speed is 0.1 mm/s. Therefore, we can obtain 100 sampled data points from a single scan. Each data point stands for coil impedance consisting of resistance and reactance. The excitation frequency, which is much greater than the frequency corresponding to the standard penetration depth, should be chosen to increase detection sensitivity, as we simply focus on surface defects in this work. The excitation frequency ranges from 50 kHz to 850 kHz in step of 100 kHz.

### 4.1. Effect on Detection Sensitivity

**Figure 3:**
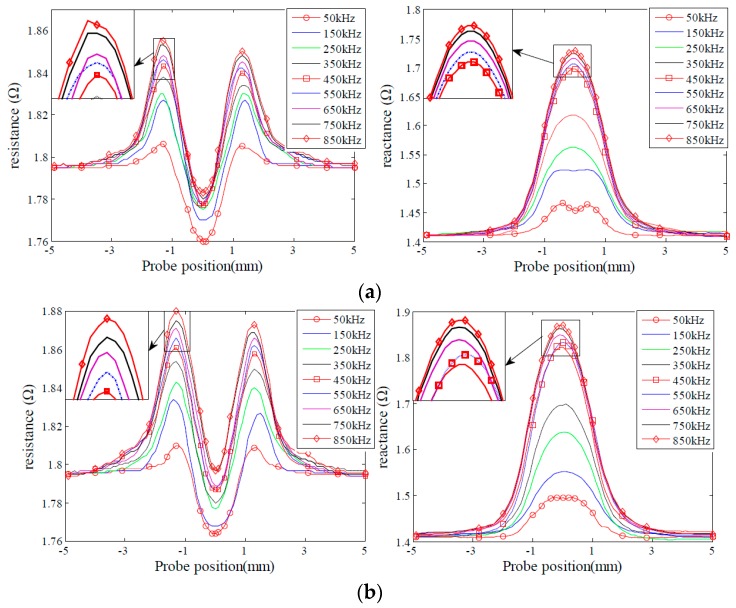

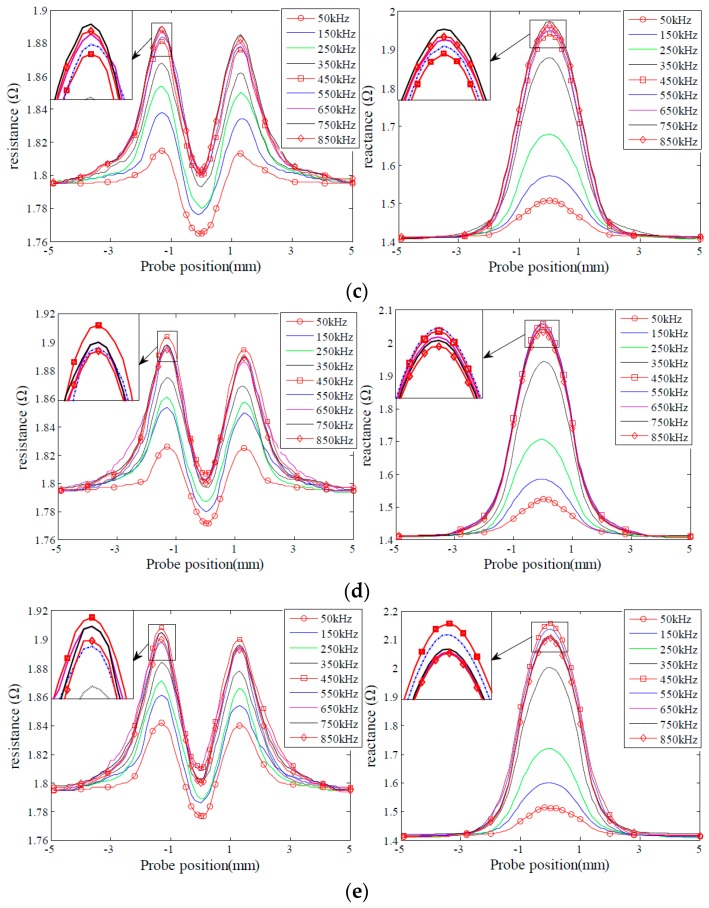
Probe signals due to the defects with different depths. (**a**) Probe signals of the defect D1; (**b**) probe signals of the defect D2; (**c**) probe signals of the defect D3; (**d**) probe signals of the defect D4; (**e**) probe signals of the defect D5.

**Figure 4:**
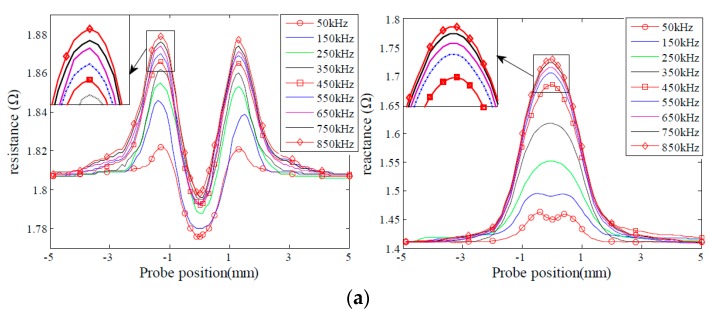

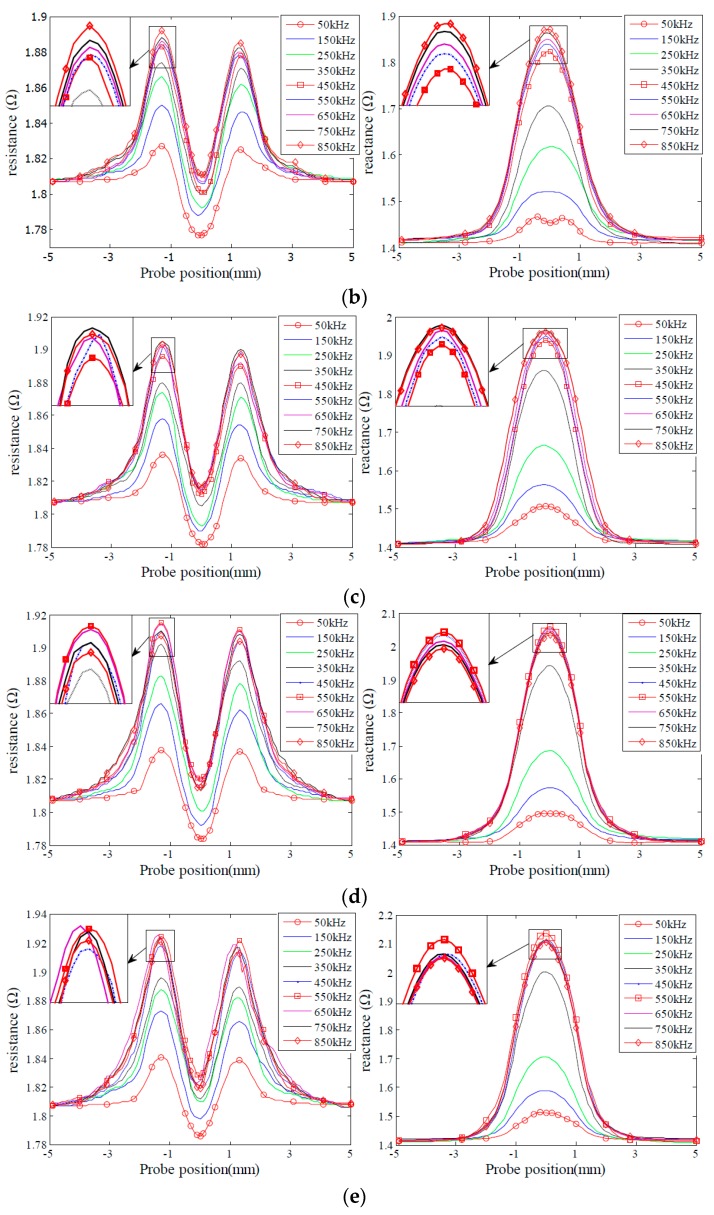
Probe signals due to the defects with different lengths: (**a**) Probe signals due to the defect L1; (**b**) probe signals due to the defect L2; (**c**) Probe signals due to the defect L3; (**d**) Probe signals due to the defect L4; (**e**) Probe signals due to the defect L5.

In order to analyze the effect of excitation frequency on defect sensitivity, experiments were carried out to acquire probe signals due to the given sets of the defects varying in depth and length, as shown in Figure 3 and Figure 4. The excitation frequencies are 50 kHz, 150 kHz, 250 kHz, 350 kHz, 450 kHz, 550 kHz, 650 kHz, 750 kHz and 850 kHz, respectively. It is observed that for the defects D1, D2, L1 and L2, both the resistance and reactance signals increase when the excitation frequency increases, and peak at 850 kHz. By contrast, for the defects D4 and D5, the probe signals increase initially, then reach a peak at 450 KHz, and decrease afterwards when the excitation frequency increases from 50 kHz to 850 kHz. Probe signals from the defects L4 and L5 possess similar characteristics to those from the defects D4 and D5. However, probe signals from the defects L4 and L5 reach a maximum at a frequency of 550 kHz. The observed phenomenon could be confirmed by the findings in [31,34] as well.

Further analysis shows that the excitation frequency, at which maximum probe signals are collected, should vary with the size of defects. For example, the probe signals due to the defect D1 increase with the increase of the excitation frequency from 50 kHz to 850 kHz. This indicates that maximum probe signals for the defect D1 shall be observed when the excitation frequency is higher than 850 kHz. In contrast, the probe signal due to the defect D5 reach maximum at the frequency of 550 kHz. This finding implies that we cannot simultaneously acquire all the maximum probe signals for different size defects at a single frequency. We also note that probe signals change significantly before reach of the peak, and decrease slightly afterwards.

### 4.2. Effect on the Contrast of Defect Features

**Figure 5:**
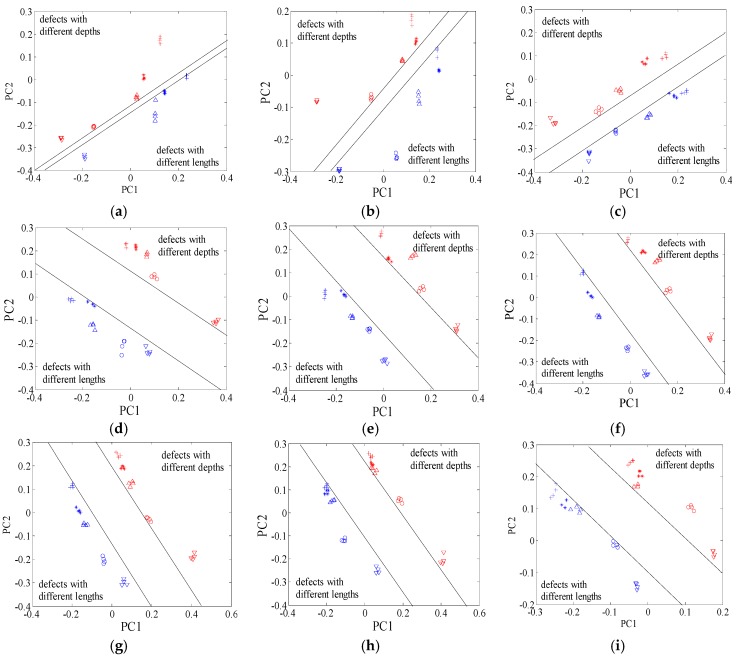
Defect features under different frequencies. (**a**) 50 kHz; (**b**) 150 kHz; (**c**) 250 kHz; (**d**) 350 kHz; (**e**) 450 kHz; (**f**) 550 kHz; (**g**) 650 kHz; (**h**) 750 kHz; (**i**) 850 kHz.

**Figure 6:**
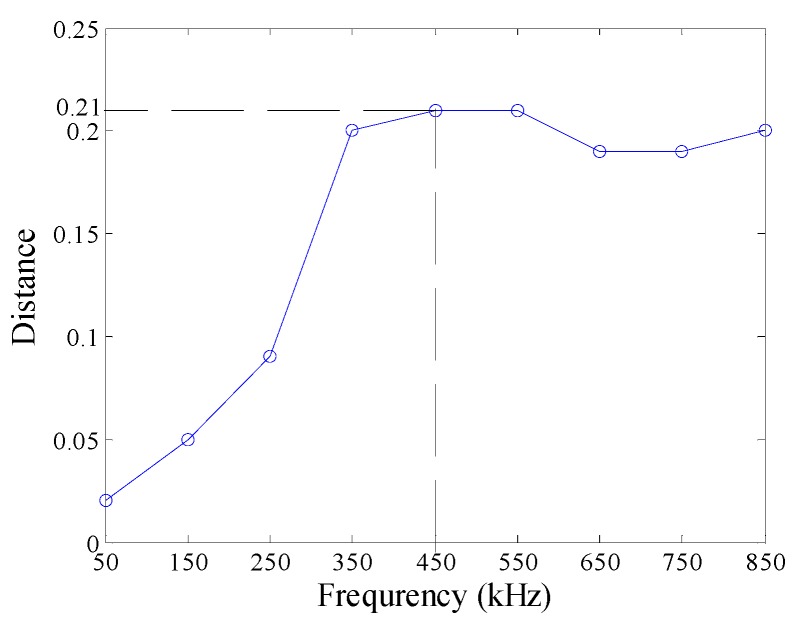
Distance between the defect features.

In this subsection, we aim at disclosing the influences of excitation frequency on the defect features. With the KPCA presented in Section 2, the defect features for identification were extracted from the probe signals, as shown in Figure 5. When excitation frequency increases from 50 kHz to 850 kHz, the margins between different type defects increase dramatically before 350 kHz, then remain almost constant, and decrease at 650 kHz, 750 kHz and 850 kHz, as shown in Figure 6.

**Figure 7:**
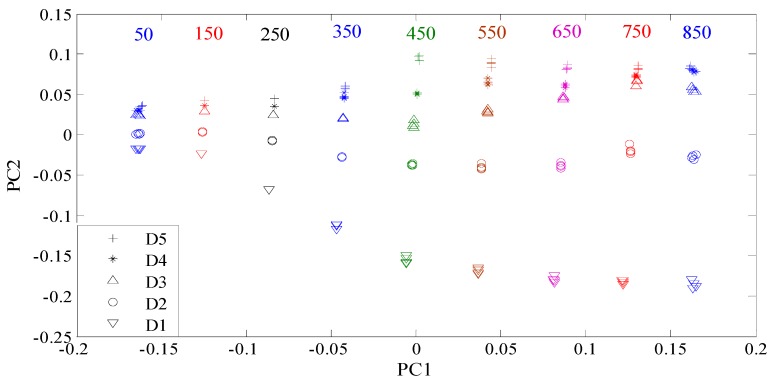
Features of the defects with different depths.

**Figure 8:**
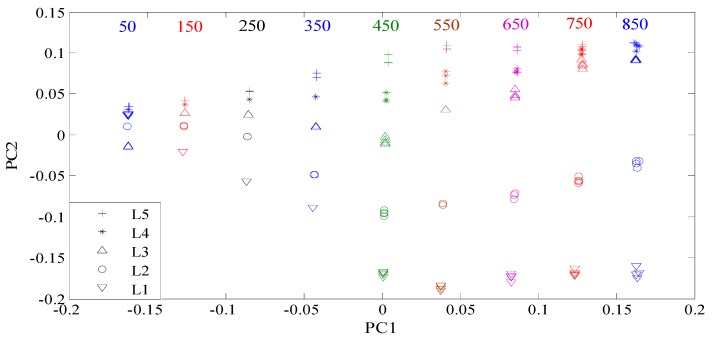
Features of the defects with different lengths.

Here, all the manufactured defects are divided into two groups. Group 1 consists of the defects with different depths, while group 2 is composed of the defects with different lengths. All the features were reprocessed using the KPCA method. The features for group 1 and group 2 were shown in Figure 7 and Figure 8, respectively. From Figure 7, it can be seen that the contrast between the defect features for group 1 increases with the increase of the excitation frequency from 50 kHz to 450 kHz. When the frequency is higher than 450 kHz, the contrast between the features of the defects D1 and D2 continues increasing. Nevertheless, the differences between the features of the defects D3 and D4 and those of the defects D4 and D5 become smaller. Similar characteristics are also observed in Figure 8. For classification, the goal is to increase the contrast between the defect features according to the structural risk minimization principle [37,38], because large contrasts between different type defects correspond to good generalization capabilities and low misclassification probabilities. For the defects in group 1, the classification accuracy should increase gradually when the excitation frequency ranges from 50 kHz to 450 kHz. However, when the frequency is higher than 450 kHz, the classification accuracy is supposed to decrease. The analysis on the defects with different depths, which will be examined and confirmed in the following subsection, applies to defects with different lengths. In summary, the optimal contrasts between the defect features would be achieved if the excitation frequency is tuned at or near the frequency at which the maximum response from the largest defect is obtained.

### 4.3. Effect on Classification Accuracy

In this subsection, we focus on the classification accuracy at different frequencies. At first, a SVM classifier was used to evaluate the accuracy when excitation frequency ranges from 50 kHz to 850 kHz. Then, the findings from the evaluation were validated by an artificial neural network (ANN) classifier.

The key to the use of an SVM classifier is to determine the penalty factor *C* and select an appropriate kernel function, which has great influence on the performance of an SVM classifier. The penalty factor *C* represents the penalty for misclassification. It controls the trade-off between the achievements of training and testing errors, which can generalize the classifier of unseen data. The larger the penalty factor *C*, the greater the penalty for training errors. In other words, we risk losing the generalization properties of an SVM classifier if we increase the penalty factor *C* too much, because the SVM classifier will try to fit as much as possible for all the training points. Currently, there are linear, polynomial and radial basis function (RBF) kernels for selection [39]. Many reports show that the RBF kernel function produces the best average performances [40]. In order to achieve optimal performances and avoid the over-fitting problem, we adopted a cross-validation technique, which is a model validation technique to assess how the results of a statistical analysis will generalize to an independent data set, to optimize the penalty factor *C* and parameters of RBF kernel function. In this work, the penalty factor *C* was set 0.00097.

Using the extracted features with KPCA, the SVM classifier was trained on the training dataset, and subsequently evaluated on the testing dataset. The classification results are listed in Table 2. It was found that the total classification accuracy increases with the increase in excitation frequency. The total classification accuracy arrives at 100% at the frequencies of 350 kHz, 450 kHz and 550 kHz, and decreases at the frequencies of 650 kHz, 750 kHz and 850 kHz. The defects D1, D2, L1 and L2 can be fully recognized at all the frequencies used. The defects D4 and D5 were identified 100% at the frequencies of 450 kHz, 550 kHz and 650 kHz. The defects L4 and L5 were correctly identified at frequencies ranging from 350 kHz to 650 kHz. In summary, the misclassifications are mainly contributed by the defects D4, D5, L4 and L5. The influences of excitation frequency on classification accuracy agree well with those on detection sensitivity and contrasts between the defect features. In short, the lowest misclassification would be achieved if the exciting frequency is tuned at or near the frequency at which the maximum response is obtained for the deepest or longest defect.

To confirm the influences on classification accuracy, ANN and SVM classifiers combined with PCA and KPCA methods were adopted at all frequencies, as shown in Table 3. The results demonstrate that the highest classification accuracy was obtained at the frequencies of 450 kHz and 550 kHz. It is noted that the maximum responses were retrieved for the deepest defect D5 at near the frequency of 450 kHz and for the longest defect L5 near the frequency of 550 kHz.

### 4.4. Discussions and Limitation

As described in the previous section, the performance of surface defect classification would be the optimal if we employ the optimum frequency at which the probe signals reach the maximum for the largest defect. For a single defect, the theoretical evidence of the existence of peak frequency corresponding to maximum probe signals could be derived from the references [31,34]. The peak frequency varies with defect size. However, the results of this work show that there exists an optimal frequency for a given set of defects from the perspective of characterization performances. The optimal frequency is extremely complicatedly connected to many factors such as probe parameters and sample properties which are dependent on temperature and stress. Moreover, it varies with the geometrical parameters of the largest defect. However, the largest defects in different cases are different. Consequently, we are currently unable to present a definite mathematical equation to calculate the optimal frequency. However, the optimal frequency could be easily determined by means of experiments. Therefore, experiment-based procedures are preferred to determine the optimal frequency, which are described as below

(1) Determine the range of the defects to be identified before inspections;
(2) Manufacture a sample defect with a maximum depth and length;
(3) Observe probe signals when excitation frequency is adjusted continuously until maximum signals are retrieved for the fabricated sample defect;
(4) The optimal frequency should be equal to the frequency corresponding to the observed maximum probe signals.

The optimal frequency should be the frequency or near the frequency at which the maximum probe signal is retrieved for the largest defect. It is noted that the findings in this work only apply to characterization of surface defects.

Part of the research activity has been concentrated on the development of novel excitations, such as multiple sinusoidal signals, pulse and chirp signals [41,42]. Compared with a single sinusoidal signal, the multi-frequency, pulse and chirp excitations aim to provide more information for better defect characterization. Searching for optimal excitation frequency could be considered part of excitation strategy optimization, as multi-frequency, pulse and chirp excitations consist of several signal sinusoidal signals from a frequency domain perspective. Therefore, determination of optimal excitation frequency should obviously contribute to the performance improvement for multi-frequency, pulse or chirp excitations as well.

Scanning angles are supposed to change probe signals significantly, thus leading to degradation of defect characterization [43]. To our knowledge, an operator will estimate the defect direction with experience or prescan calibration such that the scanning route shall be perpendicular to the defect. In addition, some researchers have carried out investigations on the influences of different scanning angles on probe signals. The simulation and experimental results demonstrate that probe signals change with the variation of scanning angle [44,45]. However, few reports are dedicated to compensating for the effect of different scanning angles for the improvement of defect classification, which means this is still an open problem to be solved. To the authors' knowledge, the eddy current imaging technique is a potential candidate to handle the effect of variant scanning angles, which is scheduled to be part of our future work.

## 5. Conclusions

In this paper, experiment-based procedures have been proposed to determine an optimal frequency over a group of defects with conventional eddy currents. This work is characterized by frequency optimization with respect to characterization performances rather than only detection sensitivity. In addition, this work features frequency optimization over a set of defects and not for a single defect.

For enhancement of surface defect characterization, the influences of excitation frequency have been identified in terms of detection sensitivity, contrasts between defect features and classification accuracy. When excitation frequency was equal to or near the frequency at which maximum probe signals were obtained for the largest defect to be inspected, probe signals from a set of defects were most sensitive on the whole. Defect features were extracted from probe signals using the KPCA algorithm. It was found that the retrieved defect features were evenly spaced, and the margins between them were the largest. According to the structure risk minimization (SRM), which is a statistical learning theory concept, the largest margins imply the lowest misclassification probabilities. Consequently, the SVM classifier, which operates on SRM, trained by the extracted features shall possess greater generalization capabilities. The experiments confirmed the best characterization performances at the selected optimal frequency, which strengthens the motivations behind the proposed techniques.

It should be noted that this paper is confined to surface defects. Future research will concentrate on determination of optimal excitation frequency for subsurface defects and the eddy current imaging technique to eliminate signal changes resulting from variant scanning angles.

## Figures and Tables

**Table 1:** Parameters of the defects.

Defects		Length (mm)	Width (mm)	Depth (mm)
different lengths (Sample 1)	L1	4	1	2.5
	L2	6		
	L3	8		
	L4	10		
	L5	12		
different depths (Sample 2)	D1	20	1	0.5
	D2			1.0
	D3			1.5
	D4			2.0
	D5			2.5

**Table 2:** Classification accuracy for each defect at different frequencies.

Defect	50 kHz	150 kHz	250 kHz	350 kHz	450 kHz	550 kHz	650 kHz	750 kHz	850 kHz
D1	100%	100%	100%	100%	100%	100%	100%	100%	100%
D2	100%	100%	100%	100%	100%	100%	100%	100%	100%
D3	80%	100%	100%	100%	100%	100%	90%	70%	90%
D4	60%	70%	100%	90%	100%	100%	100%	80%	60%
D5	60%	60%	100%	100%	100%	100%	100%	70%	70%
L1	100%	100%	100%	100%	100%	100%	100%	100%	100%
L2	100%	100%	100%	100%	100%	100%	100%	100%	100%
L3	90%	100%	100%	90%	100%	100%	100%	90%	80%
L4	60%	70%	100%	100%	100%	100%	100%	70%	80%
L5	60%	60%	90%	100%	100%	100%	100%	60%	60%
Total	88%	92%	96%	100%	100%	100%	96%	92%	92%

**Table 3:** Classification accuracy for different classifiers at different frequencies.

Classifier	Accuracy								
	50 kHz	150 kHz	250 kHz	350 kHz	450 kHz	550 kHz	650 kHz	750 kHz	850 kHz
PCA-ANN	90%	93%	95%	96%	95%	97%	94%	93%	92%
PCA-SVM	88%	88%	92%	96%	100%	100%	96%	92%	88%
KPCA-ANN	93%	95%	97%	98%	98%	98%	98%	96%	95%
KPCA-SVM	88%	92%	96%	100%	100%	100%	96%	92%	92%
